# Acute aortoiliac thrombosis and mitral valve regurgitation as acute onset of eosinophilic granulomatosis with polyangiitis in a 26-year-old patient

**DOI:** 10.1016/j.jvscit.2024.101515

**Published:** 2024-04-26

**Authors:** Luca Galassi, Giulia Lerva, Davide Passolunghi, Giovanni Marchetto, Maria Rosa Pozzi, Valerio Stefano Tolva

**Affiliations:** a School of Vascular and Endovascular Surgery, University of Milan, Milan, Italy; b Vascular and Endovascular Surgery Unit, ASST Grande Ospedale Metropolitano Niguarda, Milan, Italy; c Cardiac Surgery Unit, IRCCS San Gerardo Dei Tintori, Monza, Italy; d Rheumatology Unit, IRCCS San Gerardo Dei Tintori, Monza, Italy

**Keywords:** Acute arterial thrombosis, Churg-Strauss syndrome, Mitral valve regurgitation

## Abstract

We present a rare case of eosinophilic granulomatosis with polyangiitis (EGPA), involving a 26-year-old woman with a history of asthma and nasal polyps. The patient presented with acute aortoiliac thrombosis and mitral insufficiency, which was successfully treated with thrombolysis, aortic thromboendarterectomy, and valve replacement. Peripheral hypereosinophilia with eosinophilic infiltration of the heart led to the diagnosis of antineutrophilic cytoplasmic antibody–negative EGPA. Treatment with prednisone and mepolizumab was started, resulting in a positive outcome. This case showcases an unusual manifestation of EGPA with large size vessel involvement and requiring surgical and pharmacological treatment. It also highlights the importance of early detection for timely intervention and an improved prognosis.

Eosinophilic granulomatosis with polyangiitis (EGPA) is a rare systemic small vessel vasculitis with a reported incidence ranging from 10.7 to 13 cases per 1 million inhabitants in the general population.^1^^,^^2^ We report a case of acute aortic thrombosis and subsequent acute mitral regurgitation in a patient whose clinical, laboratory, and anatomic pathological data led to the diagnosis of an atypical presentation of EGPA. The patient was successfully treated with superior mesenteric artery thrombolysis, aortoiliac thromboendarterectomy, and mitral valve replacement. The patient provided written informed consent for the report of her case details and imaging studies.

## Case report

A 26-year-old woman was referred to our emergency department due to an 8-hour history of increasing distal lower extremity rest pain, intermittent episodes of bilateral forefoot paresthesia, and minimal sensory loss. Her vital signs and body temperature were normal. She had a history of Graves disease and nasal polyposis associated with bronchial asthma and turbinate hypertrophy. Furthermore, 3 months prior, she had been hospitalized for acute pericarditis and idiopathic pneumonia with nonfixed ground glass infiltrates.

**Fig 1:**
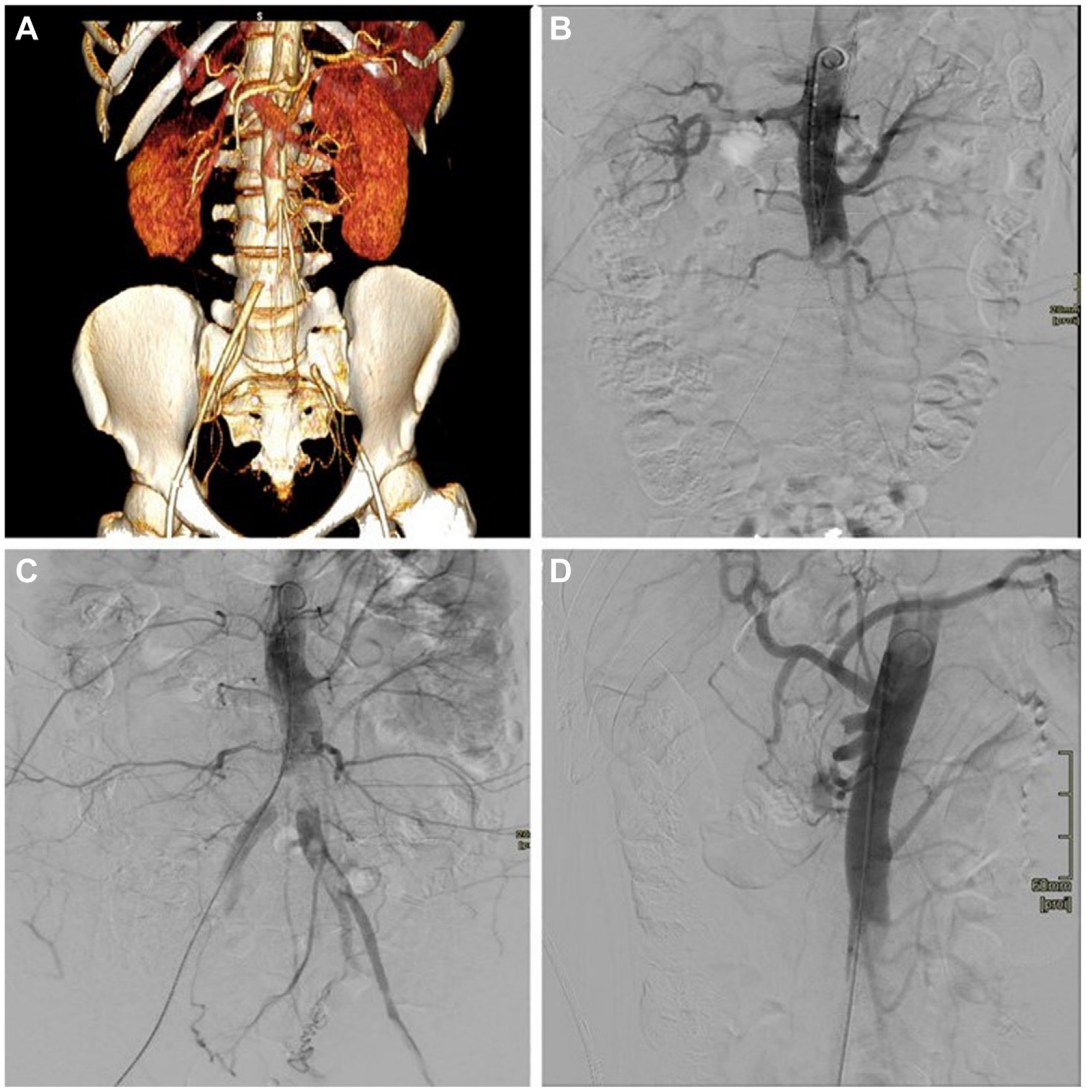
**A,** Three-dimensional reconstruction of computed tomography angiogram at first emergency department visit. **B,** Angiogram showing visceral and aortoiliac thrombosis. **C,D,** Final angiogram showing residual renal, mesenteric, and iliac thrombosis.

On clinical examination, the absence of peripheral pulses of both lower limbs was noted. Duplex ultrasound showed bilateral monophasic post-stenotic Doppler waveforms in the femoral district with no arterial flow below the knee and an ankle brachial index of 0.8. Computed tomography angiography demonstrated complete thrombotic occlusion of the abdominal aorta extending from the inferior mesenteric artery to the right common iliac artery and left external iliac artery. Complete thrombosis of the origin of the superior mesenteric artery (SMA) and partial subocclusive thrombosis of the right renal artery were also noted, despite no signs of visceral or kidney injury (Fig 1, *A*). No specific periaortic or aortic wall signs of inflammation were noted; however, significant hypereosinophilia (4800 ×10^9^ cells/L), mild neutrophilia (12,000 ×10^9^ cells/L), and elevated C-reactive protein (35 mg/dL) were observed. Transthoracic echocardiography demonstrated moderate mitral valve insufficiency compatible with rheumatic degeneration, associated with mild aortic valve stenosis.

Based on the clinical findings, our patient’s young age, and the extension of the disease, a total endovascular percutaneous approach was considered appropriate (Fig 1, *B*). Ultrasound-accelerated thrombolysis using the EkoSonic endovascular system (EKOS Corp) was attempted. Moreover, an overnight infusion of recombinant tissue plasminogen activator and systemic administration of 25,000 UI/24 hours of unfractionated sodium heparin was initiated.

The 24-hour follow-up angiography showed partial aortic recanalization with significant residual stenosis of the left external iliac artery and origin of the SMA (Fig 1, *C* and *D*). The partial thrombosis of the origin of the right renal artery appeared unmodified. Recanalization of the SMA was performed sequentially using thrombus aspiration with the Penumbra Indigo system (Penumbra Inc) and thrombectomy with an Embotrap (Cerenovus) 6.5 × 45 mm stent retriever system. The completion angiogram showed persistent thrombosis of the origin of the SMA. No further endovascular treatment was deemed appropriate, and the patient was scheduled for surgical revascularization.

**Fig 2:**
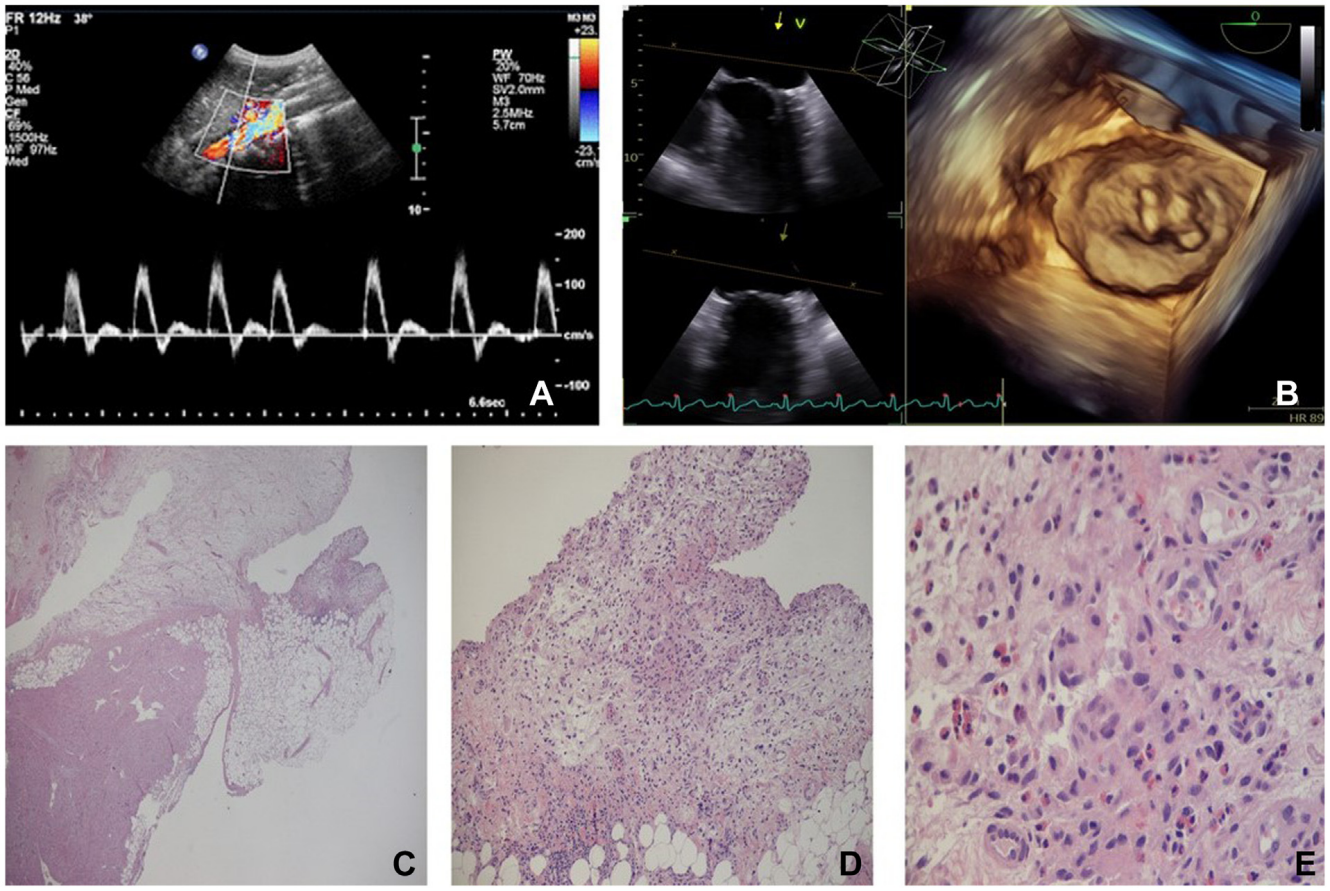
**A,** Color Doppler ultrasound of superior mesenteric artery (SMA) after surgery. **B,** Cardiac Doppler echocardiography at readmission. **C-E,** Eosinophil infiltration in the histological sample from the right atrial appendage.

Aortic embolectomy was performed through a longitudinal infrarenal incision. Given the absence of macroscopic signs of aortic wall degeneration or atherosclerosis, we opted for primary closure with a Teflon reinforcement strip, which provided suture additional support and reduced bleeding.^3^^,^^4^ Direct AMS thrombectomy was also performed and complete mesenteric and peripheral vessels revascularization obtained. Duplex ultrasound examination of visceral and iliac arteries showed triphasic Doppler waveforms following surgery (Fig 2, *A*). The postoperative course was uneventful, and the patient was discharged after 8 days.

However, she was readmitted 1 month later for progressive shortness of breath, fever, hypotension, tachycardia, and oxygen desaturation. Blood test results showed persistent hypereosinophilia, elevated C-reactive protein, and increased pro-B type natriuretic peptide (Table).

**Table:** Results of blood tests at readmission

Test	Result	Reference range
WBC count, ×10^3^/μL	23.5	4-11.0
Neutrophil count, ×10^3^/μL	9.1	2.5-8.0
Lymphocyte count, ×10^3^/μL	3.1	1.5-7.0
Monocyte count, ×10^3^/μL	2.7	1.0-4.0
Eosinophil count, ×10^3^/μL	8	0.05-0.5
Basophil count, ×10^3^/μL	0.6	0.025-0.1
PLT count, ×10^3^/μL	400	142-450
ESR, mm	45	<20
Glucose, mg/mL	105	70-100
Urea, mg/mL	32	20-45
Creatinine, mg/mL	0.95	0.72-1.05
CRP, mg/dL	19	<0.5
Hemoglobin, g/dL	11	10.5-13.5
ALT, U/L	35	10-34
AST, U/L	20	10-45
NT-proBNP, pg/mL	10,250	<125
aPTT, seconds	25	25-36
PT, seconds	12	10-13
Fibrinogen, mg/dL	480	130-330

*ALT,* Alanine transaminase; *aPTT,* activated partial thromboplastin time; *AST,* aspartate transaminase; *CRP,* C-reactive protein; *ESR,* erythrocyte sedimentation rate; *NT-proBNP,* N-terminal prohormone of brain natriuretic peptide; *PLT,* platelet; *PT,* prothrombin time; *WBC,* white blood cell.

A chest radiograph revealed signs of bilateral pulmonary congestion consistent with pulmonary edema. Transthoracic echocardiography showed a dilated left ventricle with an ejection fraction of 60% and significant progression of mitral valve regurgitation (Fig 2, *B*). Due to the progressive hypoxemic respiratory failure, veno-venous extracorporeal membrane oxygenation was initiated.

The patient underwent urgent mitral valve replacement the following day. Histological examination of the right atrial appendage showed subacute pericarditis with focal hypereosinophilia (Fig 2, *C-E*). The postoperative course was unremarkable. Based on her clinical presentation and persistent hypereosinophilia, an underlying hypereosinophilic vasculitis was suspected and investigated with a dosage of serum primary systemic vasculitis autoantibodies.^5^ Despite the absence of detectable serum antibodies, the patient was classified as being affected by eosinophilic granulomatosis with polyangiitis in accordance with the 2022 Classification Criteria for Antineutrophil Cytoplasmic Antibody-Associated Vasculitis. The criteria met by the patient included obstructive airway disease, nasal polyps, a blood eosinophil count >1 ×10^9^/L, and extravascular eosinophilic predominant infiltration.^6^

Due to the reproductive age of the patient, treatment with prednisone 25 mg twice daily, a subcutaneous injection of mepolizumab 300 mg every 4 weeks, an anticoagulant (unfractionated heparin), and aspirin 100 mg daily was started.^7^ The patient was discharged from the hospital after 15 days. The 3-month follow-up was negative for recurrence, and her eosinophil count had returned to normal.

## Discussion

EGPA is a rare multisystem autoimmune disorder mostly affecting small to medium size vessels. Although large vessel involvement has been previously reported,^8^ to the best of our knowledge, to date, there has been no report of EGPA-related massive aortic thrombosis leading to acute aortoiliac disease. EGPA is a progressive disease that can eventually lead to an increased risk of arterial thromboembolic manifestations, due to the development of progressive granulomatous necrotizing vasculitis.^9^, ^10^, ^11^

Hyperexpression of eosinophil-derived factors, such as eosinophil cationic protein, membrane basic protein, and eosinophil peroxidase,^12^^,^^13^ has been associated with an inhibitory effect on multiple levels of the natural anticoagulant pathways.^14^^,^^15^ Moreover, negative perinuclear antineutrophil cytoplasmic antibody EGPA is associated with higher eosinophilic tissue infiltration, resulting in a higher risk of thrombosis.^16^^,^^17^ Cardiac valvular involvement is still rarely observed in EGPA patients; however, mitral and tricuspid regurgitation are the most commonly reported.^18^ An early diagnosis of vasculitis might be extremely important in the clinical course of the disease. Additionally, prompt and proper medical therapy can prevent progression to a more severe stage.^19^ In fact, a retrospective analysis of the patient's medical history shows that asthma, hypereosinophilia, allergies, nonfixed lung infiltrates, and nasal polyposis were present before hospitalization.

To date, no consensus has been reached regarding the most effective strategy to manage acute aortic vasculitis-related large vessel thrombosis.^20^^,^^21^ Despite the absence of large case series exploring outcomes, endovascular procedures might carry fewer risks by avoiding extensive manipulation of potentially inflamed aortic tissue.^22^^,^^23^

However, open surgery should be chosen in the case of large vessel acute thrombosis with a high risk of distal embolization or after failure of endovascular treatment.^24^ In our case, in an urgent setting and facing extensive disease, we first opted for less invasive treatment. Subsequently, considering the residual disease extension and our patient's fitness for surgery, we chose an open approach as rescue therapy.

## Conclusions

The clinical presentation of acute systemic EGPA is extremely variable and can involve large vessels, including the aorta. Our case underscores the importance of a timely diagnosis, considering its possible unusual clinical appearance. A prompt diagnosis and tailored management could be crucial in preventing severe complications, including major thrombotic events and cardiac involvement.

The authors acknowledge support from the University of Milan through the APC initiative.

## Disclosures

None.
